# Regulation of the Orphan Nuclear Receptor *Nr2f2* by the DFNA15 Deafness Gene *Pou4f3*

**DOI:** 10.1371/journal.pone.0112247

**Published:** 2014-11-05

**Authors:** Chrysostomos Tornari, Emily R. Towers, Jonathan E. Gale, Sally J. Dawson

**Affiliations:** UCL Ear Institute, University College London, London, United Kingdom; Universitat Pompeu Fabra, Spain

## Abstract

Hair cells are the mechanotransducing cells of the inner ear that are essential for hearing and balance. POU4F3 – a POU-domain transcription factor selectively expressed by these cells – has been shown to be essential for hair cell differentiation and survival in mice and its mutation in humans underlies late-onset progressive hearing loss (DFNA15). The downstream targets of POU4F3 are required for hair cell differentiation and survival. We aimed to identify such targets in order to elucidate the molecular pathways involved in hair cell production and maintenance. The orphan thyroid nuclear receptor *Nr2f2* was identified as a POU4F3 target using a subtractive hybridization strategy and EMSA analysis showed that POU4F3 binds to two sites in the *Nr2f2* 5′ flanking region. These sites were shown to be required for POU4F3 activation as their mutation leads to a reduction in the response of an *Nr2f2* 5′ flanking region reporter construct to POU4F3. Immunocytochemistry was carried out in the developing and adult inner ear in order to investigate the relevance of this interaction in hearing. NR2F2 expression in the postnatal mouse organ of Corti was shown to be detectable in all sensory epithelia examined and characterised. These data demonstrate that *Nr2f2* is a direct target of POU4F3 *in vitro* and that this regulatory relationship may be relevant to hair cell development and survival.

## Introduction

POU4F3 is a member of the POU family of transcription factors that regulate a wide array of neuroendocrine developmental pathways [1]. This family is characterised by the presence of a bipartite DNA binding domain known as the POU domain which comprises a POU-homeodomain and a POU-specific domain separated by a linker [2]. All of these components are required for sequence-specific DNA binding [3], [4].

Though known to be expressed in the retina, spinal cord, dorsal root ganglia and Merkel cells, the investigation of POU4F3 expression and function has focused on its role in the development and maintenance of hair cells – the sensory cells of the inner ear [5]–[8]. Homozygous *Pou4f3* mutant mice demonstrate deafness and balance deficits due to the failure of nascent hair cells – cells that express hair cell markers – to develop into morphologically recognisable hair cells and subsequent death of these cells [6]–[9]. Furthermore, heterozygous mutation of human *POU4F3* is the cause of autosomal dominant late-onset progressive hearing loss (DFNA15) in several families [10]–[13].

As the molecular pathways involved in differentiation and maintenance of hair cells are largely unknown, identification of the downstream targets of POU4F3 would greatly improve current understanding of the transcription factors and target genes that mediate hair cell differentiation and maturation. Such an understanding may also aid in the explanation of the mechanism by which *POU4F3* mutation causes hearing loss in humans.

To date, the only known direct targets of POU4F3 are BDNF, NT-3 and Caprin-1 [14], [15], though the expression of other genes is strongly associated with POU4F3 expression [14], [16], [17]. In previous work we identified Caprin-1 as a direct target of POU4F3 in a subtractive hybridization screen that was carried out in UB/OC-2 cells – a conditionally immortal cell line, derived from the developing mouse inner ear sensory epithelium, that displays expression of hair cell markers [18]– that were manipulated to differentially express POU4F3 [15]. The Caprin-1 5′ flanking sequence contains POU4F3 binding sites which mediate repression of Caprin-1 expression by POU4F3 [15]. We showed that Caprin-1, which is known to promote stress granule formation [19]–[21], is expressed in both the hair cells and supporting cells of the organ of Corti as well as being involved in the hair cell response to stress [15].

The same subtractive hybridization screen also returned the orphan thyroid nuclear receptor *Nr2f2* as a putative target of POU4F3. *Nr2f2* is an orphan steroid/thyroid hormone nuclear receptor which is expressed in a range of organs in the developing embryo [22] as well as adult tissues [23]. Its most essential roles in development are in angiogenesis, heart development and remodelling the primitive capillary plexus into large and small microcapillaries; as demonstrated by knocking out the *Nr2f2* gene in mice. This change results in lethality at around E10 that is likely to be due to haemorrhage and oedema in the brain and heart [24]. Further study of the function of NR2F2 in the vascular system has revealed its importance in designating venous identity via its suppression of the Notch signalling pathway. In addition to its previously reported roles in generation of venous identity, these studies show a role for NR2F2 in cell fate determination [25].

Despite having a general role in embryogenesis, NR2F2 also demonstrates more specific actions in organogenesis. In the brain, it regulates cell migration [26]; it is essential for correct stomach patterning [27]; and has been proposed to be involved in dorso-ventral patterning of the mammalian retina [28]. Both NR2F2 and its closely related family member NR2F1 have been shown to be variably expressed in the developing mouse inner ear. NR2F2 is expressed in the distal tip of the elongating cochlear epithelium at E10 to E13.5; homogeneous expression is seen across cell types of both the greater and lesser epithelial ridges (GER & LER) in the apical coil at E15.5; and predominantly LER expression is seen in the apical-to-middle coil at the same age [29].

In contrast to NR2F2, NR2F1 expression in the developing inner ear between E14.5 and E15.5 becomes more confined to the GER in the apical cochlea. In the basal cochlea, its expression extends to the LER and the level of expression appears to decrease [29]. This decrease in basal expression correlates with the wave of hair cell differentiation from base to apex [30]. Consistent with a role in cochlear development, *Nr2f1* ^−/−^ mice display shortened cochlear ducts with an increased number of hair cells in the mid-to-apical cochlear turns; an anomaly which appears consistent with a role in Notch regulation of organ of Corti differentiation [31].

Though NR2F2 has been implicated in a number of developmental processes, its expression is known to be maintained in postnatal vertebrates. Post-natal NR2F2 expression is perhaps best-characterised in the liver where it is known to downregulate the apolipoprotein A1 (apoA1) gene [32]. However, its expression is maintained in the adult mouse brain and in a wide array of postnatal human tissue types, suggesting additional uncharacterised roles in cell maintenance [23], [33].

In this paper, we present evidence for the direct regulation of *Nr2f2* by POU4F3. Furthermore, we show that NR2F2 expression is maintained postnatally in the mouse cochlear sensory epithelium. These data add to our understanding of the downstream effects of POU4F3 signalling and widen the array of possible functions of NR2F2 to the postnatal inner ear.

## Materials and Methods

### Subtractive hybridization

POU4F3-regulated genes were identified as previously described [15]. Briefly, two populations of UB/OC-2 cells [18] were created. In one population, POU4F3 expression was increased by stable transfection of a *Pou4f3* expression construct. In the other, POU4F3 expression was reduced by stable transfection of an antisense *Pou4f3* construct. cDNA was prepared from these two cell populations and used in a subtractive hybridization screen using the PCR-Select cDNA Subtraction kit (Clontech). Differentially expressed cDNA sequences identified in this analysis were verified by a series of hybridization experiments with cDNA from the original analysis and virtual northern blot experiments with cDNA from transiently transfected cells to increase the stringency of the analysis [15]. Clones that displayed differential expression were selected for further analysis.

### BLAST analysis

Following identification of differentially expressed transcripts by subtractive hybridization, cloned cDNA was subjected to Sanger sequencing. This was followed by BLASTN analysis of transcript sequences against the ENSEMBL mouse cDNA database (NCBI m35) with a ‘near-exact match’ stringency. Matches were confirmed by alignment of clone sequence to the cDNA sequence in the ENSEMBL database using the BioEdit software package [34].

### *Nr2f2* 5′ flanking region Genomatix analysis

The Genomatix Gene2Promoter software [35] was used to identify predicted *Nr2f2* promoters for analysis. The promoter that corresponded to the transcript that best matched the *Nr2f2* transcript identified in the BLAST analysis was selected for further investigation (GXP_158616). To identify putative POU4F3 binding sites in this predicted promoter, the selected region was interrogated using the Genomatix MatInspector [36], [37] and ModelInspector [38] programs. Binding sites for analysis were selected on the basis of search stringency, proximity to the predicted transcriptional start site and promoter architecture at the relevant site. Two predicted binding sites were selected for functional analysis and were named POU recognition element-1 (PRE1) and POU recognition element-2 (PRE2).

### Electrophoretic mobility-shift assay (EMSA) analysis

Both *in vitro* translated protein and UB/OC-2 cell nuclear protein extract were used. *Pou4f3* was cloned into the pGEM-T Easy vector (Promega) under the control of the T7 or SP6 promoter and *in vitro* translated protein was generated using either the TNT-T7 Coupled Reticulocyte Lysate System (Promega) or the TNT-SP6 Quick Coupled Transcription/Translation System (Promega).

Double-stranded probe oligonucleotides were labelled in a standard T4 kinase reaction with γ^32^P γATP (GE Healthcare) using 50 ng probe per reaction. Assay reactions containing 10 µl 2x Parker buffer (16% Ficoll, 40 mM HEPES at pH 7.9, 100 mM KCl, 2 mM EDTA at pH 8.0 and 1 mM DTT), 3 µg poly(dI·dC), 2–4 µl nuclear protein extract or *in vitro* translated protein and 0–1000 ng non-radiolabelled competition probe were made up to a total volume of 20 µl and incubated on ice for ten minutes. Assay reactions were subsequently incubated at room temperature for 15–30 minutes following the addition of 1 ng labelled probe.

Reactions were loaded onto a 4% polyacrylamide (29∶1), 0.25x TBE gel and electrophoresed in 0.25x TBE at 200 V for one to three hours at 4°C. The polyacrylamide gel was subsequently dried and autoradiographs were produced by exposure of dried gels to X-Ray film at −80°C or room temperature for varying durations.

### Luciferase assays

The 4.2 kb-*Nr2f2*-Luc *Nr2f2* 5′ flanking region luciferase reporter construct was kindly provided by Dr M Vasseur-Cognet [39]. The PRE1-Luc and PRE2-Luc POU recognition element reporter constructs were cloned in our laboratory by inserting a single copy of PRE1 and PRE2 into the pGL4.23 [*luc2*/minP] reporter construct (Promega). The Dreidel expression construct was created by subcloning the Dreidel sequence from a pHM6 vector, kindly provided by Professor K Avraham [40], into pSi (Promega).

For transient transfections, ND7 cells [41] were plated at approximately 2×10^5^ cells/well density in six-well plates and cultured at 37°C with 5% carbon dioxide in L-glutamine-containing L-15 media supplemented with 10% heat-inactivated foetal bovine serum, 0.32% sodium bicarbonate, 0.25% glucose and 0.85% penicillin-streptomycin. The next morning, the culture medium was changed to DMEM with 10% heat-inactivated foetal bovine serum for at least one hour prior to transfection. Cells were transfected using a standard calcium precipitation method [42] with 100–200 ng reporter construct, 1–3 µg expression vector and 10 ng pRL-null vector (Promega). The total amount of DNA used per transfection was kept constant using pSi (Promega). Cells were then incubated for a minimum of six hours followed by washing and returning to normal culture media. Cells were harvested at least 24 hours later and assayed using the Dual-Luciferase kit (Promega).

### Site-directed mutagenesis

The 4.2 kb-*Nr2f2*-Luc-Mut reporter construct was generated by overlap extension PCR [43] using the following mutagenic primers (mutated bases underlined): PRE1 forward mutagenic primer, CTTTTTAGCCGATTTGATCACTTTGATT; PRE1 reverse mutagenic primer, AATCAAAGTGATCAAATCGGCTAAAAAG; PRE1 forward flanking primer, AAGCCTCCGGGTCGGGCCCGGAG; PRE1 reverse flanking primer, TCCGCGCTCCGGGGTCCAC; PRE2 forward mutagenic primer, GATAAAGTTGAGAGGAATTTATTTTAATTGCAGGGTAACAATGAGGTGAAGTCTGGTGTT; PRE2 reverse mutagenic primer, AACACCAGACTTCACCTCATTGTTACCCTGCAATTAAAATAAATTCCTCTCAACTTTATC; PRE2 forward flanking primer, GCTTAATGAATTCCCATCACTTGC; PRE2 reverse flanking primer, GGAATTCTCACAATCAACTAGCGG. The purified mutated fragment of PRE2 was cloned into 4.2 kb-*Nr2f2*-Luc, replacing the wild type sequence. The purified mutated fragment of PRE1 was cloned into this plasmid to create a double-mutant 4.2 kb-*Nr2f2*-Luc-Mut. Further subcloning was carried out to correct presumed PCR errors though a c to t missense mutation remained 288 bp downstream from the intended PRE1 mutagenesis site. The double-mutant probe was used in luciferase assays as described above.

### Transcription factor binding site evolutionary conservation analysis

The ECR Browser [44] was used to navigate to the region of the human genome (build “hg19”) that corresponded to the mouse sequence for PRE1 (CTTTTTAGCATATTTGATCACTTTGATT) or PRE2 (GGAATTTATTTTAATTGCATCATAACAATGAGGTGA). The view obtained was expanded to include approximately 70 bp of flanking sequence and adjusted to include only mouse, rat and chimpanzee alignments. This view was submitted to the Mulan software and the phylogenetic tree obtained was not adjusted. The results of the Mulan analysis were submitted to MultiTF using the ‘vertebrates’ and ‘optimised for function’ settings against the TRANSFAC Professional Version 10.2 library to identify evolutionarily conserved POU domain transcription factor binding sites. Positional weight matrices (PWMs) for POU-domain transcription factors were included in this analysis as POU family binding sites are clearly related [4] despite there being subtle sequence-specific differences in the recognition elements of different POU4 family members [45]: OCT1_B, OCT1_Q5, OCT1_Q6, OCT4, OCT_C, OCT_Q6, POU1F1_Q6, POU3F2 and POU6F1.

### Cryosectioning and immunocytochemistry

Adult (i.e. >P21) C57BL/6J mice, adult Sprague Dawley rats and P1 C57BL/6J mice were killed in accordance with Schedule I of the UK Animals (Scientific Procedures) Act (1986). This study was approved by the UCL Biological Services Ethical Review Committee. Inner ears were isolated and the apical bone of the cochlea was perforated to facilitate fixation in 4% paraformaldehyde solution in PBS for one to two hours at room temperature or overnight at 4°C. Cryoprotection was carried out by incubation of samples in 30% sucrose in PBS at 4°C overnight. Tissue was mounted in 1% low gelling agarose (Sigma-Aldrich) in PBS which had been heated to 140°C and allowed to cool to 37°C. Once set, a block was cut, attached to a cryostat chuck with OCT compound and rapidly frozen by immersion in liquid nitrogen prior to cryosectioning at −20 to −25°C. Sections of 15 µm were cut using a CM1850 cryostat (Leica).

Prior to immunolabelling, sections were permeabilised and blocked for one hour at room temperature with blocking solution (10% goat serum in PBS) containing 0.1–0.5% Triton-X. Slides were then incubated in blocking solution with an antibody raised against NR2F2 (a kind gift from Dr M Studer) 1∶1000 at 4°C overnight [26]. Following three five-minute PBS washes, 2 µg/ml Alexa 488-conjugated anti-rabbit secondary antibody, 1 Unit/ml Alexa 633-conjugated Phalloidin and 5 µM DAPI in PBS or blocking solution was added for two hours at room temperature. Following three further five-minute PBS washes, samples were mounted in Fluoromount G and visualised using a Zeiss LSM 510 Meta microscope. Relative expression comparisons were made between apical to basal cochlear turns within individual slides.

## Results

### Identification of *Nr2f2* as a putative target of POU4F3

In order to identify target genes of POU4F3 that may be involved in hair cell survival and maintenance, a subtractive hybridization was carried out in UB/OC-2 cells followed by serial virtual Northern analysis as described previously [15]. One putative upregulatory target of POU4F3 identified by this screen was Clone D8. This clone was sequenced and subjected to BLAST analysis against the ENSEMBL mouse cDNA database (based on NCBI m35 data) to identify known mouse cDNA sequences to which it corresponded. Clone D8 matched the 3′ region of the related *Nr2f1* and *Nr2f2* cDNA sequences which are highly homologous in this region. However, the match to *Nr2f2* (ENSMUST00000032768) was more extensive (343 bp, 100% sequence similarity: p = 8.6×10^−273^) than the match to *Nr2f1* (116 bp, 87% sequence similarity: p = 3.3×10^−25^). Therefore, this analysis identified *Nr2f2* as an upregulated transcript from the POU4F3 forward subtractive hybridization library.

As *Nr2f2* was identified as a putative upregulatory target of POU4F3 in the subtractive hybridization screen, its expression in UB/OC-2 cells was investigated as these cells constitutively express POU4F3 in their proliferating state and are derived from the mouse inner ear at E13, i.e. after the onset of NR2F2 expression in the developing ear [18], [29]. *Nr2f2* mRNA and protein were found to be present in proliferating UB/OC-2 cells by reverse transcriptase PCR, western blot and immunohistochemistry (Figure S1). NR2F2 expression was localised to UB/OC-2 cell nuclei, consistent with its reported subcellular localisation [26], [29].

### POU4F3 binds two sites in the *Nr2f2* 5′ flanking region identified by Genomatix software analysis

For POU4F3 to directly regulate a gene, it must bind to the target gene promoter via a sequence specific binding site. To establish whether *Nr2f2* is a direct or indirect target of POU4F3 we performed bioinformatic and functional analysis of the *Nr2f2* 5′ flanking region to identify any such binding sites. The Genomatix MatInspector and ModelInspector programmes were used to interrogate the most proximal 5 kb of the predicted *Nr2f2* promoter (GXP_158616, identified by the Gene2Promoter software) [35]–[38].

**Figure 1:**
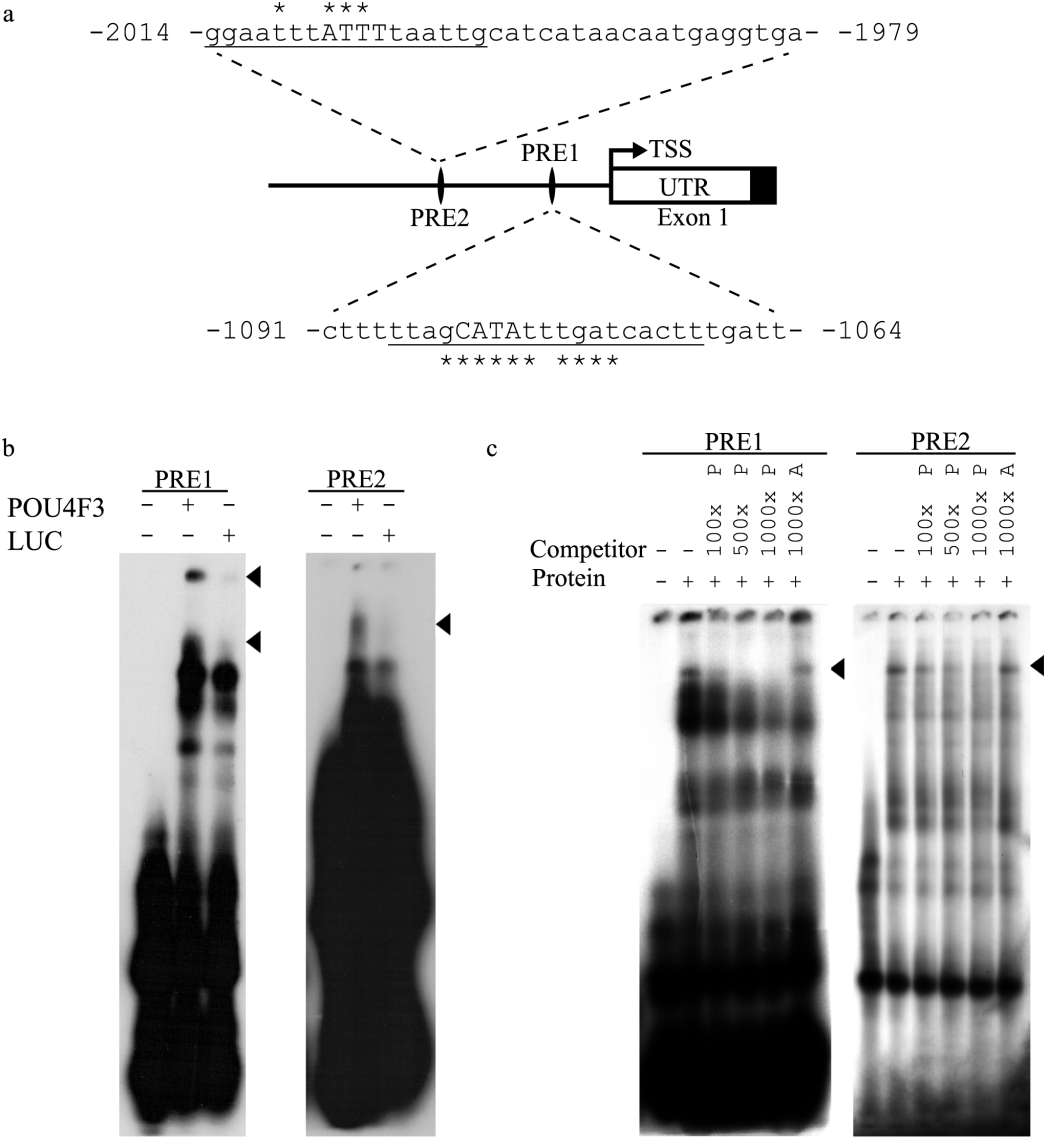
Identification and verification of POU4F3 recognition elements in the *Nr2f2* 5′ flanking region. *a*. Schematic diagram of the location of two putative POU4F3 recognition elements (PREs) identified in the *Nr2f2* 5′ flanking region and used in EMSA analysis. Underlined bases correspond to POU4F3 binding sites predicted by MatInspector software with capital letters denoting matches to the core sequence. The asterisks signify matrix position conservation >60/100 (*TSS*, transcriptional start site and *UTR*, un-translated region). *b*. Sequences shown in *a* were used as radiolabelled probes in EMSA analysis with *in vitro* translated POU4F3 or a Luciferase control. Bandshifts due to protein-specific binding by POU4F3 are indicated by arrowheads. *c*. The same probes were used in EMSA analysis with UB/OC-2 cell nuclear protein extract. Reactions were incubated either alone, with UB/OC-2 cell nuclear protein extract or with the nuclear protein extract and an excess of non-radiolabelled competitor oligonucleotide as indicated. The ‘*P*’ suffix refers to non-radiolabelled POU4F3 binding sequence whereas the ‘*A’* suffix indicates a non-radiolabelled AP4 binding sequence. POU4F3-sequence-specific shifts are indicated by arrowheads.

Using MatInspector software at the highest stringency (0.05) a number of putative POU4F3 binding sites were identified. Of these, the two most proximal to the *Nr2f2* transcriptional start site were selected for further analysis and designated POU regulation element (PRE) 1 and 2 (Figure 1a). PRE2 also overlapped with a SORY OCT1 module identified using the Genomatix ModelInspector software i.e. PRE2 is within an experimentally verified functional promoter subunit that contains predicted binding sites for both OCT1 and SORY.

The ability of POU4F3 to bind these putative recognition elements was tested by electrophoretic mobility-shift assay (EMSA) analysis. *In vitro* translated POU4F3 produced bandshifts for both PRE1 and PRE2 that were not reproduced by *in vitro* translated luciferase protein (Figure 1b), demonstrating POU4F3-specific binding to PRE1 and PRE2. In addition, endogenous UB/OC-2 cell POU4F3 was shown to bind to these two sequences in EMSA competition analysis (Figure 1c). Hence, UB/OC-2 cell nuclear protein extract binding was reduced with increasing amounts of unlabelled POU4F3 consensus sequence but not with an excess of an unrelated transcription factor binding site, demonstrating the presence of POU4F3 in the shifted complex (Figure 1c).

### POU4F3 activates the 4.2 kb *Nr2f2* 5′ flanking region containing PRE1 and PRE2

**Figure 2:**
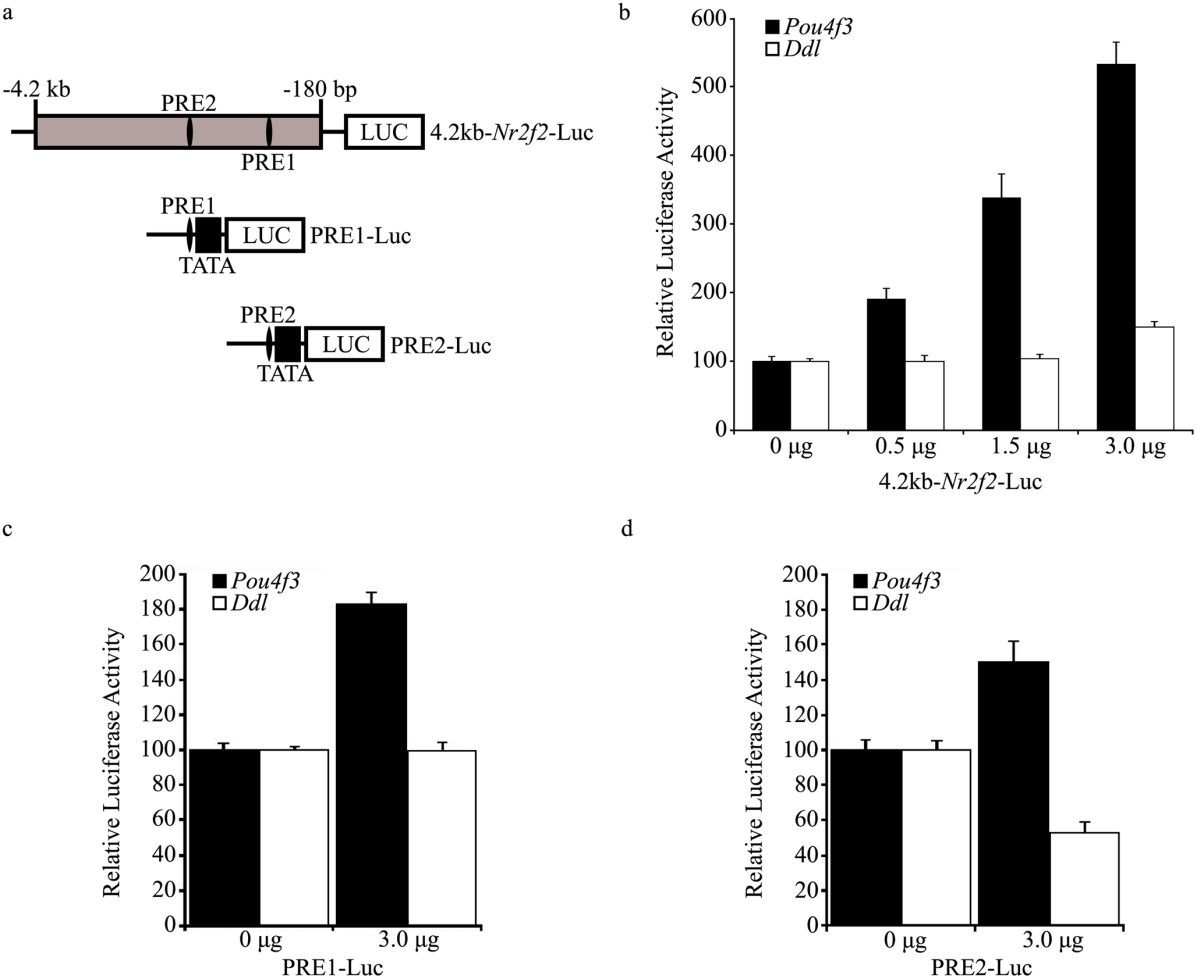
POU4F3-mediated activation of PRE1, PRE2 and a 4.2 kb *Nr2f2* 5′ flanking sequence. *a*. Schematic diagram of reporter constructs used in luciferase reporter assays in ND7 cells. POU4F3 recognition element (PRE) 1 and 2 are shown in an *Nr2f2* 5′ flanking region fragment (*grey box*) cloned upstream of a luciferase reporter gene (*LUC*). The location of this fragment relative to the *Nr2f2* transcriptional start site is shown. *b*. Evaluation of the response of 4.2 kb-*Nr2f2*-Luc to increasing levels of POU4F3 or *dreidel* mutant POU4F3 (*Ddl*) expression construct. The luciferase activity of the reporter is normalised to its response to the empty expression vector and results are expressed relative to this. *c*. Response of the PRE1-Luc reporter construct in co-transfection experiments with POU4F3 and *dreidel* expression constructs. *d*. Evaluation of the response of PRE2-Luc in similar experiments to *c*. Error bars represent the s.e.m in *b, c* and *d* (n = 6 for each data point).

Having demonstrated the ability of POU4F3 to bind to PRE1 and PRE2, we tested whether POU4F3 can regulate the *Nr2f2* 5′ flanking region that contains these sites. A luciferase reporter construct containing 4.2 kb of the *Nr2f2* 5′ flanking region (4.2 kb-*Nr2f2*-Luc, Figure 2a) was used in co-transfection studies in ND7 cells. This analysis demonstrated a dose-dependent increase in 4.2 kb-*Nr2f2*-Luc activity in response to increasing POU4F3 levels. Compared to basal activity, promoter activity was five times higher at the maximal amount of POU4F3 expression construct used (3 µg) and was not replicated with a non-DNA-binding POU4F3 mutant (*dreidel*), showing that this activation is dependent on POU4F3-specific DNA binding (Figure 2b).

These experiments were also conducted in UB/OC-2 cells but gave inconsistent results (data not shown). Experiments in this cell type may have been complicated by a number of factors. For example, proliferating UB/OC-2 cells endogenously express POU4F3 which may have been sufficiently expressed to mask the variation induced by the co-transfected POU4F3 expression construct. UB/OC-2 cells also vary their POU4F3 expression depending on differentiation status; it is, therefore, possible that POU4F3 expression varies more dynamically than is currently known. Given these possibilities, ND7 cells were used as POU4F3 activation of *Nr2f2* constructs was consistent in this assay.

To investigate whether POU4F3 activates 4.2 kb-*Nr2f2*-Luc via PRE1, PRE2 or a combination of both, each was cloned upstream of a minimal promoter in a luciferase vector to generate PRE1-Luc and PRE2-Luc (Figure 2a) and used in co-transfection studies with increasing amounts of POU4F3. In ND7 cells, co-transfection with a POU4F3 expression construct produced a 1.8-fold upregulation of PRE1-Luc (Figure 2c) and a 1.5-fold upregulation of PRE2-Luc (Figure 2d). In both cases, this activation required functional POU4F3 as *dreidel* mutant protein did not upregulate either binding site (Figure 2c & d).

### Activation of 4.2 kb-*Nr2f2*-Luc is dependent on PRE1 and PRE2

**Figure 3:**
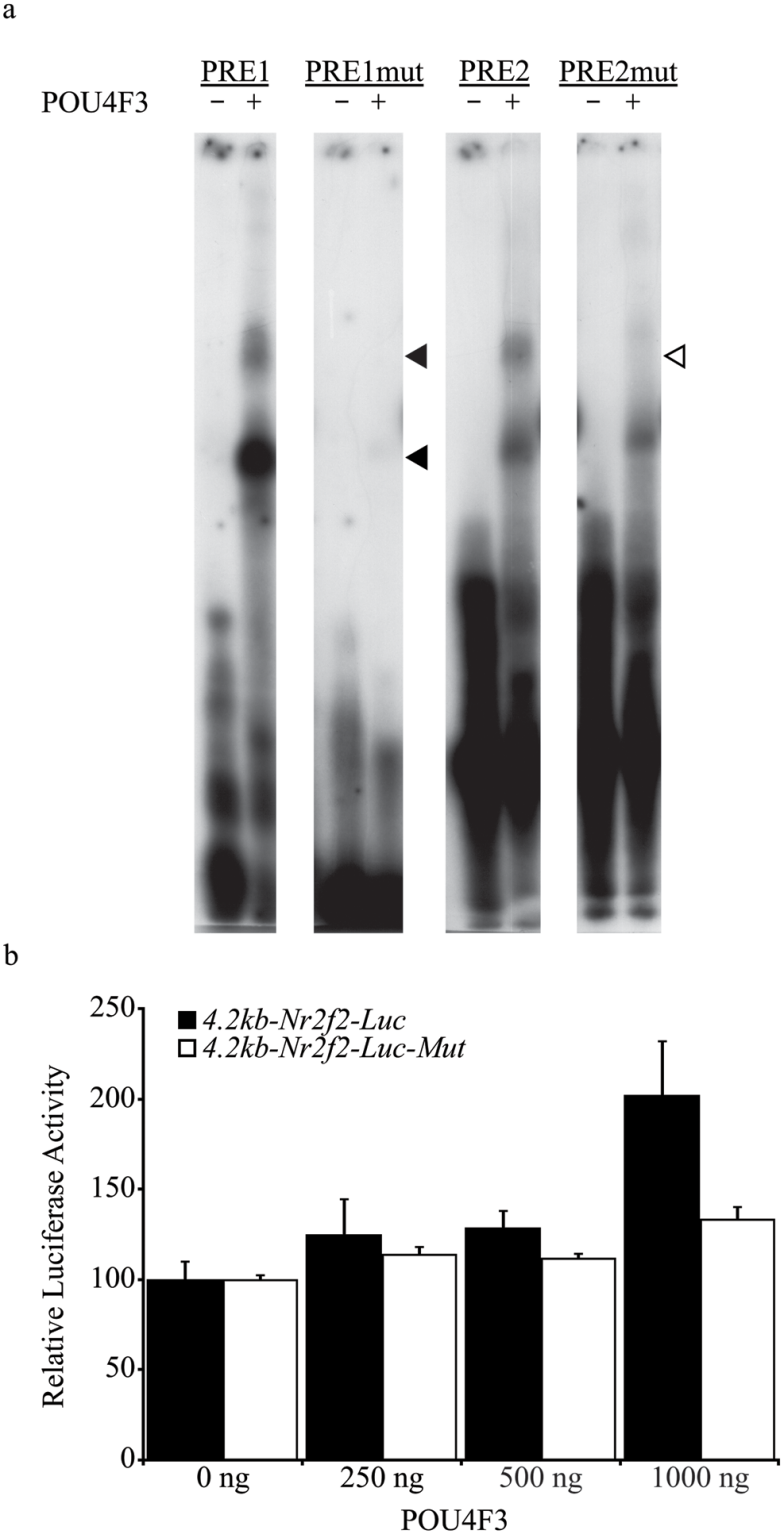
POU4F3 recognition element mutation attenuates POU4F3 binding and activation of the *Nr2f2* 5′ flanking region. *a*. EMSA analysis of POU4F3 recognition element mutations on POU4F3 binding. Probes were either incubated alone or with *in vitro* translated POU4F3. *Closed arrowheads* indicate POU4F3-specific PRE1 bandshifts and the *open arrowhead* indicates the POU4F3-specific PRE2 bandshift. The mutation of each site severely compromises the ability of POU4F3 to bind to each sequence. *b*. Luciferase reporter assay upon co-transfection of POU4F3 with the 4.2 kb-*Nr2f2*-Luc-Mut reporter construct. Error bars represent the s.e.m (n = 6 for each data point).

To confirm that POU4F3-mediated regulation of the *Nr2f2* promoter is dependent on PRE1 and PRE2, both sites were mutated to attenuate POU4F3 binding. Mutant versions of PRE1 and PRE2 were synthesised and tested in EMSA analysis which showed effective attenuation of *in vitro* translated POU4F3 binding (Figure 3a). Both mutations were then introduced into the 4.2 kb-*Nr2f2*-Luc construct by overlap extension PCR to produce 4.2 kb-*Nr2f2*-Luc-Mut and the effect of these mutations on POU4F3-specific activation was investigated in co-transfection studies. Regulation of 4.2 kb-*Nr2f2*-Luc-Mut was attenuated to 1.3-fold compared to a two-fold activation of 4.2 kb-*Nr2f2*-Luc at the maximal amount of POU4F3 expression construct used (1 µg). This suggests that PRE1 and PRE2 are required for activation of the *Nr2f2* 5′ flanking region by POU4F3 (Figure 3b).

### Evolutionary Conservation of PRE1 and PRE2

Similar to protein-coding regions of the genome, functionally important regulatory elements are conserved across species [46]–[48]. Therefore, the evolutionary conservation of the POU4F3 binding sites PRE1 and PRE2 within the *Nr2f2* 5′ flanking sequence was investigated to indicate the potential functional importance of these sites *in vivo*. The Mulan program performs an analysis of sequences from multiple species; constructs phylogenetic trees; creates alignments; and identifies evolutionarily conserved regions. The output of this analysis is used as input for the MultiTF program which identifies evolutionarily conserved transcription factor binding sites [48].

**Figure 4:**
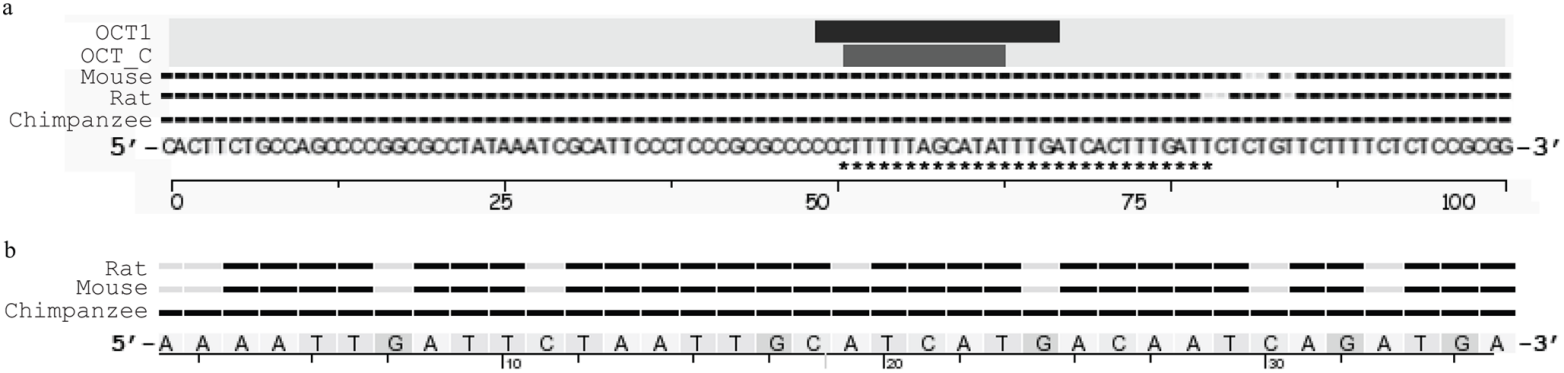
Evolutionary conservation of transcription factor binding sites in the *Nr2f2* 5′ flanking region. Schematic representation of the conservation of the region surrounding the human PRE1 sequence (*a*) and the human PRE2 sequence (*b*) compared to corresponding mouse, rat and chimpanzee sequences. *a*. The well-conserved PRE1 sequence is indicated by asterisks with evolutionarily conserved binding sites for the POU domain transcription factors OCT1 and OCT_C indicated by horizontal bars. Conserved bases are represented by black boxes with mismatches in grey. *b*. PRE2 is less well conserved with eight mismatched bases and no corresponding POU domain transcription factor binding sites in the TRANSFAC database. Conserved bases are again represented by black boxes with mismatches in grey.

The MultiTF program relies on the TRANSFAC Professional Version 10.2 library [49], however, no PWM is available for POU4F3 in this database. Therefore, PWMs for closely related transcription factor binding site families (i.e. OCT and POU) were used as POU family binding sites are closely related [4]. This analysis revealed two evolutionarily conserved binding sites in the human region of *NR2F2* that correspond to the PRE1 locus in the mouse *Nr2f2* 5′ flanking region. The overlapping binding sites identified correspond to OCT1 and OCT_C PWMs (Figure 4a). No such evolutionarily conserved binding sites were identified for PRE2 which is less well-conserved than PRE1 across the species assessed (Figure 4b). This conservation suggests that PRE1 is more likely to play an important role in the regulation of *Nr2f2* expression *in vivo* over recent evolution.

### Expression of NR2F2 in the embryonic rat inner ear

The inner ear’s function is primarily achieved by mechanosensory hair cells which are surrounded by support cells and extracellular matrix that form a complicated microanatomy which is required to achieve the special senses of hearing and balance. Accordingly, the development of this organ requires a complex co-ordination of cellular proliferation, differentiation and morphogenetic movement [50].

POU4F3 and NR2F2 are both expressed in the embryonic mouse inner ear. In the developing cochlea, *Pou4f3* expression begins in the base and extends to include all hair cells of the inner ear by birth. This expression is unique to hair cells and correlates with the wave of hair cell differentiation [6]–[8], [51]. NR2F2 is more widely expressed in the developing apical organ of Corti with its expression level gradually reducing to become more refined to the LER in the basal cochlea [29].

**Figure 5:**
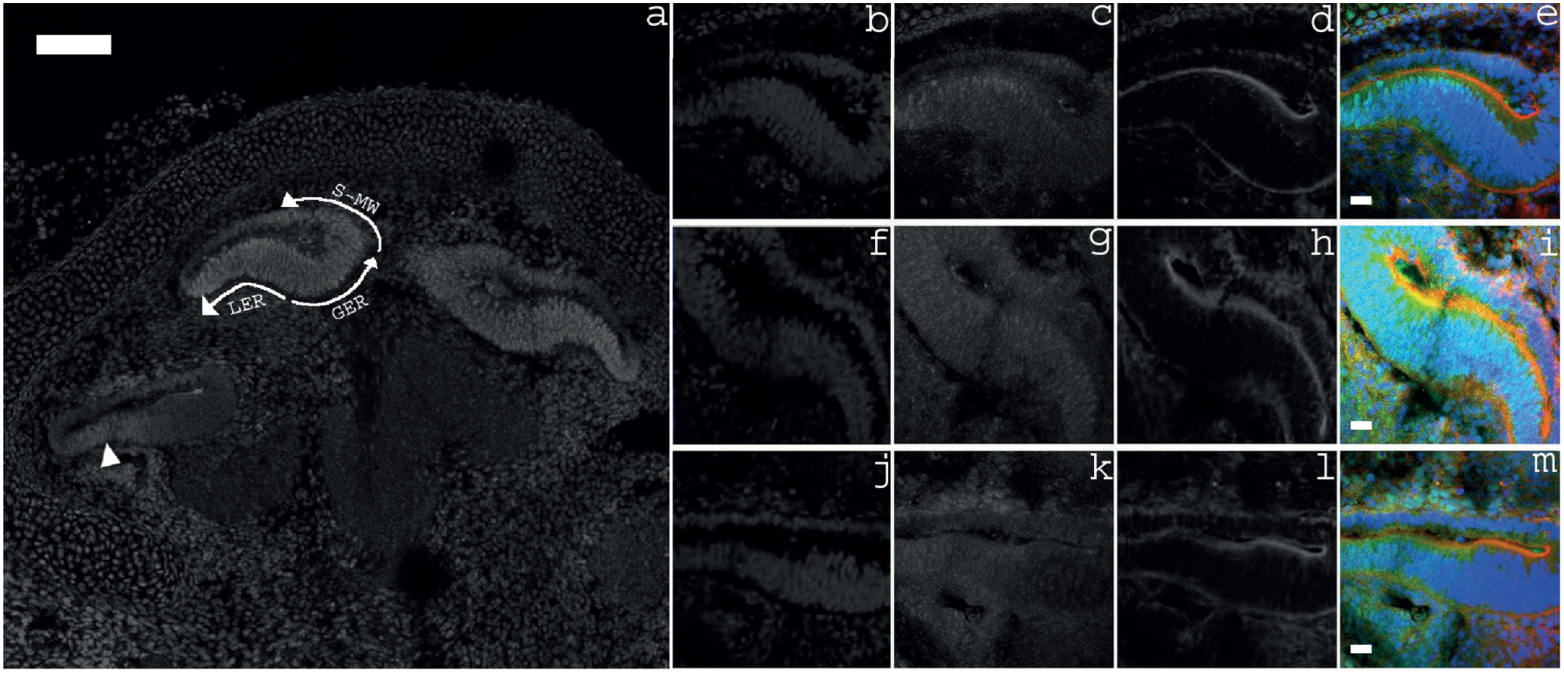
Expression of NR2F2 in the embryonic rat cochlea. *a*. Overview of NR2F2 expression throughout the cochlea at E18. NR2F2 is most strongly expressed in the cochlear duct. In the apical duct, strong expression is seen in the super-medial wall (*S-MW*), greater epithelial ridge (*GER*), and lesser epithelial ridge (*LER*). This expression is reduced to the lesser epithelial ridge in the basal duct (*arrowhead*). *b–e*. Apical cochlear duct; *f–i*. Middle cochlear duct; and *j–m*. Basal cochlear duct. *b, f & j*, cell nuclei stained with DAPI; *c, g & k*, NR2F2 labelling; *d, h & l*, filamentous actin labelled by Phalloidin; and *e, i & m,* Merge of DAPI (*blue*), NR2F2 (*green*) and Phalloidin (*red*), s*cale bars*: 100 µm in *a* and 20 µm in *e, i & m*.

The putative interaction of POU4F3 and *Nr2f2* in the inner ear was investigated by further characterising the expression profile of NR2F2 in the E18 rat inner ear. NR2F2 expression was seen throughout the developing cochlea at this age with strongest expression seen in the greater epithelial ridge, lesser epithelial ridge and supero-medial wall of the apical cochlear duct (Figure 5). Expression decreased in an apical-to-basal direction with NR2F2 expression being limited to the lesser epithelial ridge of the basal cochlear duct (Figure 5). This embryonic expression profile is in keeping with the previously reported expression profile of NR2F2 in the embryonic mouse inner ear [29].

### NR2F2 expression in the postnatal mouse inner ear

The subtractive hybridization experiment that identified *Nr2f2* as a putative target of POU4F3 was conducted to elucidate the pathway through which POU4F3 affects hair cell differentiation and survival. In the murine model, hair cell differentiation requires POU4F3 in the embryonic and early postnatal period [8]. However POU4F3 is also required for hair cell survival and is expressed postnatally [10]–[12], [17]. It must therefore influence its targets postnatally – though the targets required for hair cell maturation will not necessarily be identical to those required for maintenance. As NR2F2 is expressed postnatally in a number of organs, we investigated whether it is expressed in the postnatal inner ear to ascertain whether it is has the potential to play a role in hair cell maintenance as a POU4F3 target.

**Figure 6:**
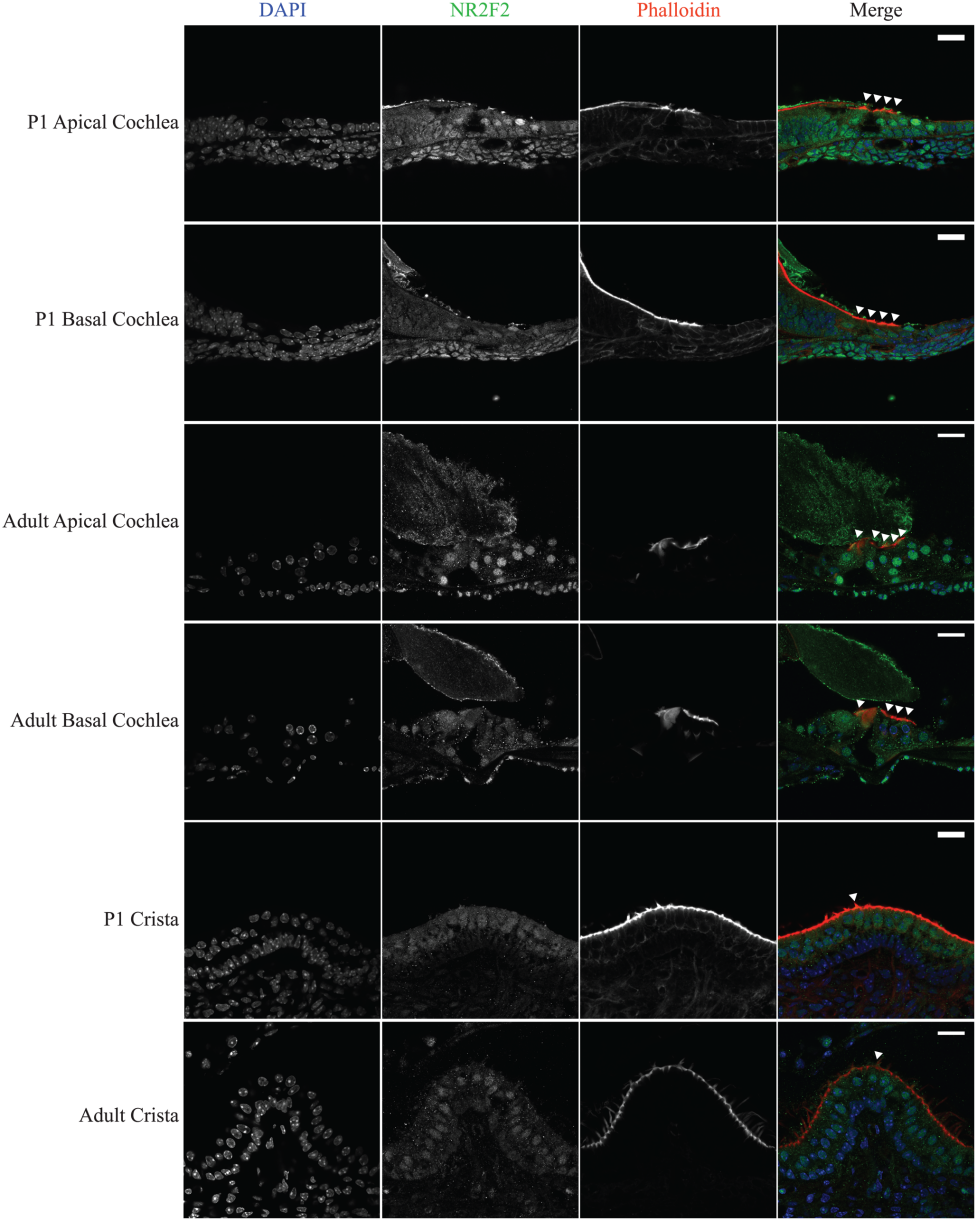
Expression of NR2F2 in the postnatal mouse inner ear. Cryotome sections of P1 and adult mouse inner ears were subjected to immunohistochemistry in order to characterise their postnatal NR2F2 expression pattern. NR2F2 labelling is seen throughout the sensory epithelia of the cochlea and cristae. In the cochlea, NR2F2 labelling is of greater intensity in the apex than the base. NR2F2 is expressed in the nuclei of apical hair cells at all ages examined. In P1 mice, NR2F2 expression is reduced in basal hair cells. In adult mice, the expression of NR2F2 in basal hair cell nuclei appears further reduced. NR2F2 is also expressed in the hair cells of the cristae of the semicircular canals and this expression is maintained into adulthood. In merged images DAPI is *blue*, NR2F2 is *green*, Phalloidin is r*ed*, a*rrowheads* indicate hair cells and s*cale bars*: 20 µm.

As in the embryonic cochlea, NR2F2 expression was widespread in the postnatal mouse cochlea both within and around the sensory epithelium. Throughout the P1 cochlea a typical nuclear pattern of NR2F2 expression was seen in hair cells as well as a number of supporting cells of the sensory epithelium i.e. Hensen cells, Claudius cells and inner sulcus cells (Figure 6). Expression appeared stronger in the apical coil including the hair cells as compared to the basal coil and hair cells. This apical-to-basal expression gradient was maintained in the adult cochlea where NR2F2 expression in the basal cochlea further decreased so that NR2F2 expression was no longer observed in the nuclei of basal hair cells. As in the cochlea, NR2F2 expression is widespread though variable in the postnatal cristae where its expression is greatest within the sensory epithelium, including hair cells (Figure 6).

## Discussion

POU4F3 expression is essential for hair cell differentiation and maintenance [6]–[8], [10]–[12]. However, it’s only known direct targets (BDNF, NT-3 and Caprin-1) and downstream targets (Gfi1 and Lhx3) do not sufficiently explain the link between POU4F3 and the hair cell phenotype [14]–[16], [40]. In this paper, we furthered the understanding of this pathway by identifying and verifying *Nr2f2* as a new regulatory target of POU4F3.

A combination of bioinformatic analysis, EMSA and reporter gene assays demonstrated that POU4F3 binds to two sites in the 4.2 kb *Nr2f2* 5′ flanking region and that the most proximal binding site (PRE1) is well-conserved through recent evolution. The sites identified were shown to be required for POU4F3-mediated upregulation of this region as their mutation attenuates this effect and were sufficient to confer POU4F3 activation upon a heterologous promoter (Figure 2). These results suggest that *Nr2f2* is a direct regulatory target of POU4F3.

### NR2F2 in the developing inner ear

Experiments in embryonic rats confirmed expression of NR2F2 in the developing cochlea (Figure 5) [29]. The widespread expression of NR2F2 indicates the presence of otic regulators of this gene. However, our data reveal that hair cell-selective *Nr2f2* expression could be regulated by POU4F3 given its hair cell-specific expression pattern in the inner ear [6], [8].

As well as the POU4F3 binding sites described here, the *Nr2f2* 5′ flanking region is known to contain response elements for retinoic acid, sonic hedgehog, and Notch [52]–[54] which are all involved in inner ear development [50], [55], [56]. Furthermore, NR2F2 is known to mediate signalling between mesenchymal and endothelial compartments and it is thought that organs which require the differentiation of mesenchyme to epithelium display NR2F2 expression in the mesenchyme [57]. Epithelial-mesenchymal interactions give rise to most of the otic capsule and retinoic acid (which interacts with NR2F2) is thought to orchestrate mesenchyme-epithelial interactions in inner ear morphogenesis [54], [57], [58]. Though their relevance in the inner ear is not yet known, NR2F2 could act as an integrator of these important developmental pathways.

### NR2F2 in the postnatal inner ear

The potential role of NR2F2 in hair cell survival and maintenance was investigated in the adult inner ear. In the postnatal mouse cochlea, NR2F2 expression is highest in the apex and falls to undetectable levels in basal hair cells. This pattern correlates with a previous report of higher apical POU4F3 expression in the postnatal rat cochlea [17]. The potentially matching expression pattern of these two transcription factors, if verified, supports the possibility that their interaction is active in the postnatal cochlea.

Apical hair cells are responsible for the detection of low frequency sounds and are the first hair cells of the cochlea to exit the cell cycle at approximately E13 in the developing mouse [59]. However, these hair cells are the last to complete their differentiation as the wave of hair cell differentiation proceeds from base to apex [30]. Though the gradient of NR2F2 expression seen in development mirrors hair cell differentiation this pattern persists despite full differentiation, i.e. after the onset of hearing. The nuclear expression pattern in these cells suggests that NR2F2 is acting as a transcription factor. However, as the postnatal role of NR2F2 is currently poorly understood, further work is required to determine its role in the hair cells of the inner ear.

The postnatal cochlear expression pattern of NR2F2 reported here is contrary to previously published data where it was not detected in the postnatal mouse organ of Corti [29]. This may be explained by several factors. For example, different antibodies were used in these experiments and tissue was obtained from different mouse strains [26], [29], [60]. As transcription factors typically have low expression levels, a small change in the sensitivity of the differing immunohistochemistry assays could account for this discrepancy.

In addition to its cochlear expression, NR2F2 expression was detected in the nuclei of hair cells and supporting cells of the postnatal mouse crista. The cristae form part of the vestibular apparatus and are required for the detection of rotational acceleration. A role for NR2F2 in this system would, therefore, imply a role in balance function. This is the first report of NR2F2 expression in the vestibular system and further work to improve the understanding of its relative expression and function at this site, as compared to the cochlea, may assist in elucidation of the molecular basis for phenotypic differences between cochlear and vestibular hair cells.

### NR2F2 function in the inner ear

Given the diversity of genes and pathways in which NR2F2 is involved, it is possible that NR2F2 could play a novel role in cochlear development and maintenance. However, despite knowledge of its regulation, the function of NR2F2 in the inner ear remains unknown. Furthermore, the complex regulation of *Nr2f2* and its various targets [61] make it difficult to differentiate any effects of POU4F3 from other activators of *Nr2f2* which are known to be important in inner ear differentiation e.g. sonic hedgehog [62]. The hair-cell-specific expression of POU4F3 suggests that its interaction with *Nr2f2* represents a regulatory mechanism that may be restricted to these sensory cells.

It is notable that a number of other reported regulatory targets of POU4F3 are transcription factors, including Lhx3 and Gfi-1 [16], [40] suggesting that it lies at the head of a complex and diverse regulatory pathway and may act as master-regulator of hair cell function. This is certainly consistent with the evidence provided from both transgenic mice with targeted deletion of *Pou4f3* and humans with *POU4F3* mutations, which exhibit loss of hearing due to loss of hair cell function.

Postnatally, mitochondrial biogenesis has been implicated in hair cell survival following the administration of ototoxic medications and noise exposure [63], [64]. NR2F2 has been shown to be involved in white adipose tissue mitochondrial biogenesis and may therefore influence this otoprotective function in postnatal hair cells [65]. Also, established roles of NR2F2 such as angiogenesis may be relevant to other cochlear cells that do not express POUF43 but do express NR2F2 [65].

Embryonic lethality at E10 makes it hard to assess the effect of *Nr2f2* knockout on the developing mouse inner ear and no relevant conditional knockout mouse exists [24]. In the adult *Nr2f2* knockout mouse, there is no overt phenotype, though hearing has yet to be assessed in these animals [66]. However, postnatal expression of NR2F2 in a number of organs – including the inner ear – suggests that it plays a functional role beyond development. Current evidence suggests that NR2F2 is most important in regeneration and de-differentiation in pathological conditions [65].

Therefore, beyond the verification of POU4F3 regulation of *Nr2f2 in vivo*, the characterisation of NR2F2 function in development and under pathological conditions in mature animals is essential to clarify the putative role of this versatile orphan nuclear receptor in the development and maintenance of hearing and balance.

## Supporting Information

Figure S1 ***Nr2f2*** **expression in UB/OC-2 cells.** *Nr2f2* expression was investigated in proliferating UB/OC-2 cells. *a*, cDNA was generated from proliferating UB/OC-2 cells and subjected to PCR which demonstrated amplification of the predicted *Nr2f2* fragment (213 bp). Integrity of the cDNA was demonstrated by amplification of *Gapdh* (450 bp) with sizes of amplified fragments measured against a 1 kb marker (*M*). Adjacent lanes have been removed for presentation purposes. *b*, The specificity of an anti-NR2F2 antibody was verified by western blot. A predominant 50 kDa band (*arrow*) with the expected molecular weight of NR2F2 is detected. Immunohistochemistry was carried out with (*c*) and without (*d,* secondary antibody alone) an anti-NR2F2 antibody in UB/OC-2 cells. This analysis showed NR2F2 expression localised to OC-2 cell nuclei. *i*, nuclei were stained DAPI; *ii*, NR2F2 expression; *iii*, Phalloidin labelling; and *iv*, shows a merged image of *i*, *ii* and *iii* with DAPI in *blue*, NR2F2 in *green* and Phalloidin in *red*. *Scale bars*: 100 µm.
(TIF)
