# Supplementary figures and images for: Regulation of the Orphan Nuclear Receptor Nr2f2 by the DFNA15 Deafness Gene Pou4f3
Source: PLoS One. 2014 Nov 5;9(11):e112247. doi: 10.1371/journal.pone.0112247 (PMC4221282; doi:10.1371/journal.pone.0112247)

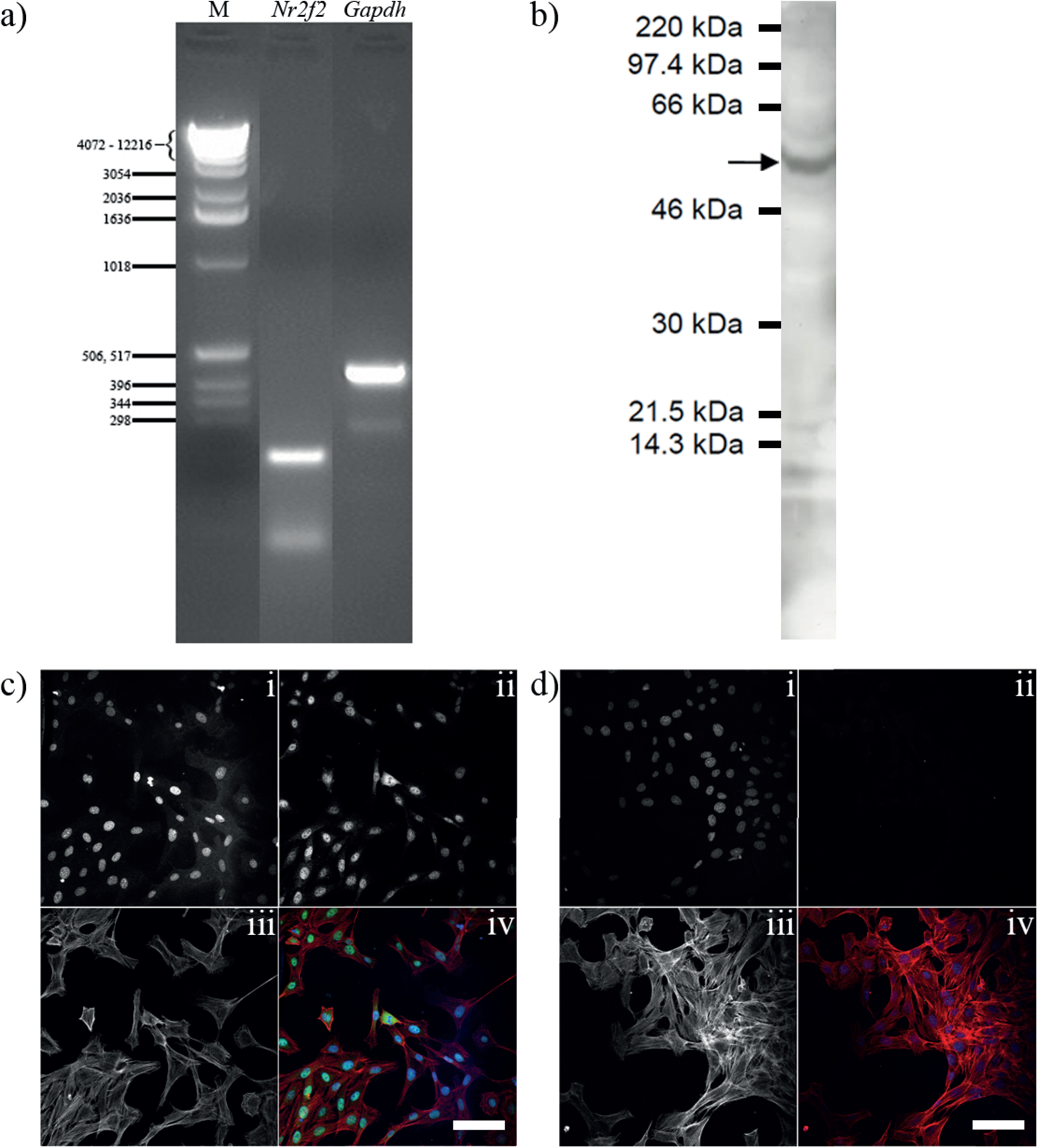

Supplement: Figure S1 — Nr2f2 expression in UB/OC-2 cells. Nr2f2 expression was investigated in proliferating UB/OC-2 cells. a, cDNA was generated from proliferating UB/OC-2 cells and subjected to PCR which demonstrated amplification of the predicted Nr2f2 fragment (213 bp). Integrity of the cDNA was demonstrated by amplification of Gapdh (450 bp) with sizes of amplified fragments measured against a 1 kb marker (M). Adjacent lanes have been removed for presentation purposes. b, The specificity of an anti-NR2F2 antibody was verified by western blot. A predominant 50 kDa band (arrow) with the expected molecular weight of NR2F2 is detected. Immunohistochemistry was carried out with (c) and without (d, secondary antibody alone) an anti-NR2F2 antibody in UB/OC-2 cells. This analysis showed NR2F2 expression localised to OC-2 cell nuclei. i, nuclei were stained DAPI; ii, NR2F2 expression; iii, Phalloidin labelling; and iv, shows a merged image of i, ii and iii with DAPI in blue, NR2F2 in green and Phalloidin in red. Scale bars: 100 µm. (TIF) [file pone.0112247.s001.tif]
